# Predicting incomplete cytoreduction in patients with advanced ovarian cancer

**DOI:** 10.3389/fonc.2022.1060006

**Published:** 2022-12-15

**Authors:** Eva K. Egger, Marie Antonia Buchen, Florian Recker, Matthias B. Stope, Holger Strunk, Alexander Mustea, Milka Marinova

**Affiliations:** ^1^ Department of Gynecology and Gynecological Oncology, University Hospital Bonn, Bonn, Germany; ^2^ Medicine Center Bonn, Medical Care Center, Bonn, Germany; ^3^ Department of Nuclear Medicine, University Hospital Bonn, Bonn, Germany

**Keywords:** ovarian cancer, cytoreduction, CT scan, residual tumor, mesenteric lymph nodes

## Abstract

**Purpose:**

The most important prognostic factor for survival in ovarian cancer patients is complete cytoreduction. The preoperative prediction of suboptimal cytoreduction, considered as any residual disease at the end of surgery, could prevent futile surgery and morbidity. Here, we aimed to identify markers in the preoperative abdominal CT scans of an unselected cohort of patients with ovarian cancer that are predictive of incomplete cytoreduction.

**Methods:**

This is a single-institution retrospective analysis of 105 epithelial ovarian cancer (EOC) patients treated with surgical cytoreduction between 2010 and 2020. Twenty-two variables on preoperative abdominal CT scans were compared to the intraoperative macroscopic findings by Fisher’s exact test. Parameters with a significant correlation between intraoperative findings and imaging were analyzed by multivariate binary logistic regression analysis regarding the surgical outcome of complete versus incomplete cytoreduction.

**Results:**

Complete cytoreduction (CC), indicated by the absence of macroscopic residual disease, was achieved in 79 (75.2%) of 105 patients and 46 (63.9%) of 72 International Federation of Gynecology and Obstetrics (FIGO) stage III and IV patients. Twenty patients (19%) were incompletely cytoreduced due to miliary carcinomatosis of the small bowel, and six patients (5.7%) had various locations of residual disease. Thirteen variables showed a significant correlation between imaging and surgical findings. Large-volume ascites, absence of numerically increased small lymph nodes at the mesenteric root, and carcinomatosis of the transverse colon in FIGO stage III and IV patients decreased the rate of CC to 26.7% in the multivariate analysis.

**Conclusion:**

Large-volume ascites, the absence of numerically increased small lymph nodes at the mesenteric root, and carcinomatosis of the transverse colon are markers in preoperative CT scans predicting a low chance for complete cytoreduction in unselected ovarian cancer patients in a real-world setting.

## Introduction

Ovarian cancer is still the most frequent cause of death in women suffering from gynecologic malignancies (1). Standard treatment is upfront surgery followed by platinum- and taxane-containing chemotherapy. In the case of the International Federation of Gynecology and Obstetrics (FIGO) stage IIIB to IVB, bevacizumab, an antivascular endothelial growth factor antibody, is added. Additionally, patients with deficient homologous recombination are treated with PARP inhibitors (2–4). Optimal cytoreduction in epithelial ovarian cancer (EOC) patients, considered as no macroscopically visible residual disease at the end of surgery, is the most important factor for survival (2, 5–7). As most patients present in the advanced stages of the disease, optimal cytoreduction will include multivisceral surgery harboring the risk of morbidity and mortality (8). As reported rates of optimal cytoreduction range between 20% and 85%, there will be patients undergoing surgery without survival benefits and patients who might profit from neoadjuvant chemotherapy before debulking surgery (5). While the specificity of contrast-enhanced abdominal computed tomography (CT) scans for the detection of peritoneal carcinomatosis is about 88%, the sensitivity is only 68% (9). By far, the most common reason for suboptimal cytoreduction is extensive small bowel mesentery or serosal carcinomatosis, often underestimated in presurgical CT scans (10–12). Therefore, an optimal preoperative screening would identify the subgroup of patients where complete cytoreduction will not be possible in an upfront situation to avoid futile surgery.

In the case of recurrence, the prospectively validated Arbeitsgemeinschaft Gynäkologische Onkologie (AGO) score identifies 75% of patients with recurrent ovarian cancer where optimal cytoreduction will be achieved again. However, no such tool is available in the primary situation (13). In the primary setting, the therapeutic sequence—upfront surgery followed by adjuvant chemotherapy or neoadjuvant chemotherapy followed by surgery—remains the key issue arising in ovarian cancer patients deemed fit enough for surgery.

Here, we aimed to identify, in a real-life cohort of patients with ovarian cancer, the group of patients least likely to undergo complete cytoreduction despite a radical multivisceral surgical approach by using radiological markers in the preoperative pelvic and abdominal CT scan.

## Material and methods

### Data collection

This study was conducted in accordance with the standards of the ethics committee of the Faculty of Medicine at the University of Bonn, Germany. The study obtained ethical approval (No 329/21) from the ethics committee of the Faculty of Medicine at the University of Bonn, Germany. Patients gave informed consent for the use of their data. The institutional record database was screened for epithelial ovarian cancer patients with cytoreductive surgery between January 2010 and December 2020. A total of 346 patients were identified. Patients with recurrent disease (n = 63) and patients with CT examinations without oral and intravenous contrast administration (n = 178) were excluded from the analysis. All CT scans were performed within a maximum of 28 days before surgery. In the case of neoadjuvant chemotherapy, two or three cycles of chemotherapy were completed before CT scan acquisition. Gastrografin was used as a radiopaque contrast medium 1 h prior to image acquisition; an intravenous contrast agent (iopamidol) was also administered. The CT scan was performed with the patients in a supine position by using a 64-slice scanner (Brilliance, Philips Healthcare, Amsterdam, the Netherlands); both arterial and portal venous phase images were acquired. Two radiologists with at least 15 years of experience in abdominal imaging blinded to the surgical details and outcome were asked to evaluate all abdominal CT scans for the following 23 items: liver metastasis, ascites, absence/presence of numerous small lymph nodes at the mesenteric root (number > 10, short diameter < 1 cm), paracolic peritoneal carcinomatosis (PC), right and left diaphragm thickening as a sign of PC, general peritoneal thickening, PC of the small and large bowel mesentery, PC of the small bowel mesenteric root, PC of the spleen, extrahepatic PC considered as PC on Glisson’s capsule, PC in the porta hepatis/hepatoduodenal ligament, PC of the gallbladder, wall thickening of the small bowel as suspected correlate of a serosal PC, PC of the rectosigmoid, PC of the transverse colon, PC of the ileocecal region, PC at the omentum minus and majus, PC on the stomach wall, pelvic tumor, and retroperitoneal infrarenal lymph node enlargement.

Surgery reports and pathologic findings were screened for carcinomatosis in all the above-mentioned regions. Ascites were measured by CT scan only, as surgery reports were too vague. The peritoneal carcinomatosis index as the sum of carcinomatosis, quantified by size in 13 regions of the abdomen, was retrospectively calculated based on surgical and pathological reports to provide information on tumor burden (14). The main criterion for optimal debulking was no macroscopically visible residual disease at the end of surgery. In all cases of incomplete resection, the location of tumor residuals was documented.

### Statistical analysis

In the first step, all variables were analyzed by Fisher’s exact test to identify significant correlations between imaging and intraoperative finding. Differences were considered to be significant at a threshold of ≤0.05. In a second step considering FIGO stage III and IV patients only, variables with a significant correlation of imaging and intraoperative finding were analyzed by multivariate binary logistic regression regarding the surgical outcome (complete or incomplete cytoreduction). The positive and negative predictive values of the CT scan were calculated for the analysis of serosal, mesenterial, and mesenteric root carcinomatosis. All statistical analyses were performed using Minitab Version 18 (Minitab LLC, State College, PA, USA).

## Results

### Baseline information

Baseline patient characteristics are shown in **Table 1**. Presurgical CT scans of 105 non-selected patients with ovarian cancer were evaluated. At surgery, complete cytoreduction (CC) was achieved in 79 (75.2%) of 105 patients and 46 (63.9%) of 72 FIGO stage III and IV patients. Twenty patients (19%) underwent incomplete cytoreduction due to miliary carcinomatosis of the small bowel; in further six patients (5.7%), the reason was tumor involvement of the porta hepatis (n = 2), liver metastases (n = 1), and spread to the retroperitoneum (n = 2) or pancreas (n = 1). Thirteen of 22 variables in total showed significant correlations between imaging and surgical findings as depicted in **Table 2**. The evaluation of the preoperative CT scans was especially difficult regarding the issue of carcinomatosis of the small and large bowel mesentery with 57 and 43 patients regarded as not evaluable. The 23rd variable “ascites”, was only evaluated on CT scans, as the surgical reports showed low accuracy regarding the three predefined conditions: 1) no ascites, 2) ascites only in the pelvis, and 3) ascites in all four quadrants of the abdomen. No ascites were seen in 51 patients, only in the pelvis in 16 patients, and ascites in all four quadrants of the abdomen were present in 38 patients.

**Table 1:** General patient characteristics.

N	105
Age	Median 53 (range 32-82)
Histology	Serous: 91/86.7% Endometrioid: 9/8.6% Mucinous: 3/2.9% Clear cell: 2/1.9%
BMI	<19: 6/5.7% 20–24: 48/45.7% 25–30: 32/30.5% 31–40: 14/13.3% >40: 5/4.8%
Cytoreduction	Complete: 79/75.2% Incomplete: 26/24.8%
Neoadjuvant chemotherapy	Yes: 54/51.4% No: 51/48.6%
Duration of surgery in minutes	Median 343 (range 126–691)
Number of erythrocyte concentrates	Median 2 (range 0–19)
Ascites	None: 51/48.6% Only pelvic ascites: 16/15.2% In all 4 quadrants: 38/26.2%
Peritoneal carcinomatosis index (PCI)	Median 9 (range 1–29)
Preoperative CA 12-5	Median 115 (range 11–9,647 U/ml)
FIGO stage	No. of patients
IA	11
IB	1
IC	9
IIA	2
IIB	6
IIC	3
IIIA	5
IIIB	4
IIIC	55
IVA	6
IVB	2

BMI, body mass index; FIGO, International Federation of Gynecology and Obstetrics.

**Table 2:** Correlation of tumor location according to CT scan and according to surgery report.

Tumor location	Imaging: tumor/no tumor	Surgery reports: tumor/no tumor	Imaging not conclusively assessable/no documented intraoperative assessment	p-Value
Paracolic	36/68	70/35	1/0	0.03
Right diaphragm	34/71	44/61	–	0.001
Left diaphragm	24/81	27/78	–	0.02
Pelvic tumor	87/18	82/23	–	0.004
Peritoneal thickening in general	78/25	30/75	2/0	0.002
Small bowel mesentery	20/28	38/67	57/0	0.001
Large bowel mesentery	20/42	38/67	43/0	<0.001
Mesenteric root	48/55	21/84	2/0	0.006
Splenic hilum	14/90	15/90	1/0	0.005
Liver surface	41/64	8/97	–	0.26
Porta hepatis	40/65	6/99	–	0.002
Gall bladder	10/78 (12× CHE)	2/91	5/0	>0.99
Infrarenal lymph nodes	104/1	30/74	0/1	>0.99
Small intestine serosa	29/73	18/87	3/1	0.08
Rectosigmoid	45/38	56/49	22/0	0.54
Transverse colon	32/69	22/83	4/0	0.002
Ileocecal pol	13/86	21/84	6/0	0.06
Stomach wall	18/86	8/97	1/0	0.003
Omental cake	33/71	52/53	1/0	<0.001
Liver metastases	4/100	4/100	1/0	0.15
Omentum minus	11/89	10/95	5/0	0.08

CHE, cholecystectomy.

**Figure 1:**
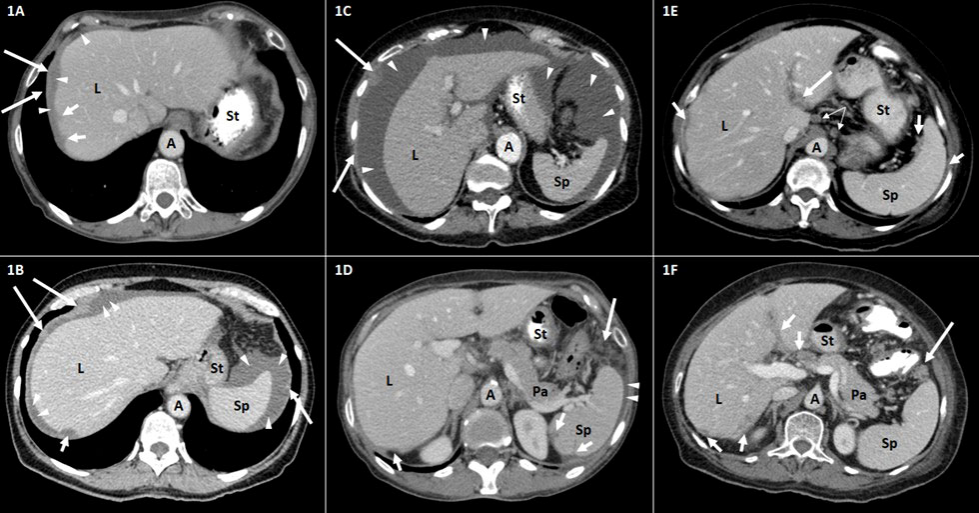
Representative CT findings in the upper abdomen in patients with advanced ovarian carcinoma FIGO stage III–IV. Axial contrast-enhanced CT scans of the upper abdomen **(A–F)**. A, aorta; L, liver; Pa, pancreas; Sp, spleen; St, stomach. **(A, B)** Tumor implants of the diaphragm (long arrows) and the liver (short arrows). The thickening of the right hemidiaphragm (long arrows) can be distinguished from surrounding perihepatic ascites (arrowheads). The surface tumor deposit at the dome of the liver (short arrows) causes scalloping of the lateral **(A)** and posterior **(B)** liver surface. **(C)** Large amount of ascites (arrowheads) in the upper abdomen. Peritoneal knotty implants (long arrows) are shown. **(D)** Tumor scalloping (short arrows) of the posterior surface of the spleen and the liver. Small amount of perisplenic ascites (arrowheads). Fat tissue stranding and tumor nodularity (long arrow) are seen in the fat adjacent to the splenic flexure of the colon. **(E)** Tumor implants (long arrow) along the hepatogastric ligament. Peritoneal thickening and tumor scalloping of the surface of the spleen and the liver (short arrows). Round suprarenal lymph nodes (narrow arrows). **(F)** Tumor implants (short arrows) in the porta hepatis along the falciform ligament and of the posterior liver surface. Tumor nodule (long arrow) is seen in the fat adjacent to the splenic flexure of the colon. FIGO, International Federation of Gynecology and Obstetrics.

**Figure 2:**
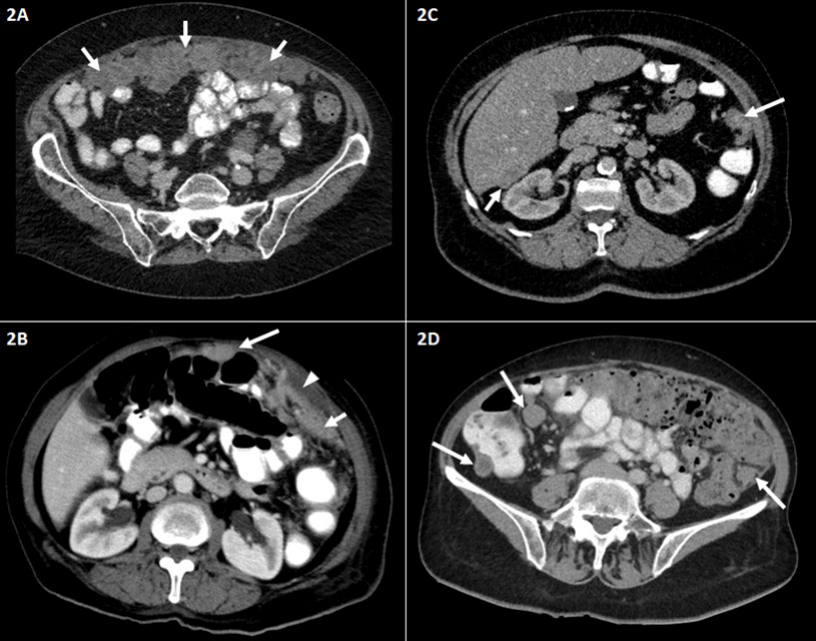
Representative abdominal CT findings in patients with advanced ovarian carcinoma FIGO stage III–IV. Axial contrast-enhanced CT scans of the middle and lower abdomen **(A–D)**. **(A)** Irregular soft tissue mass representing large omental plaques called omental cake (short arrows) common site of intraperitoneal seeding of ovarian carcinoma. **(B)** Peritoneal thickening (short arrow) and small amount of ascites (arrowhead). Peritoneal nodule adjacent to the transverse colon (long arrow). **(C)** Peritoneal nodules in the left paracolic gutter (long arrow) and the hepatorenal recess (Morison’s pouch) (short arrow). **(D)** Peritoneal implants/nodules in the right and left paracolic gutter. FIGO, International Federation of Gynecology and Obstetrics.

**Figure 3:**
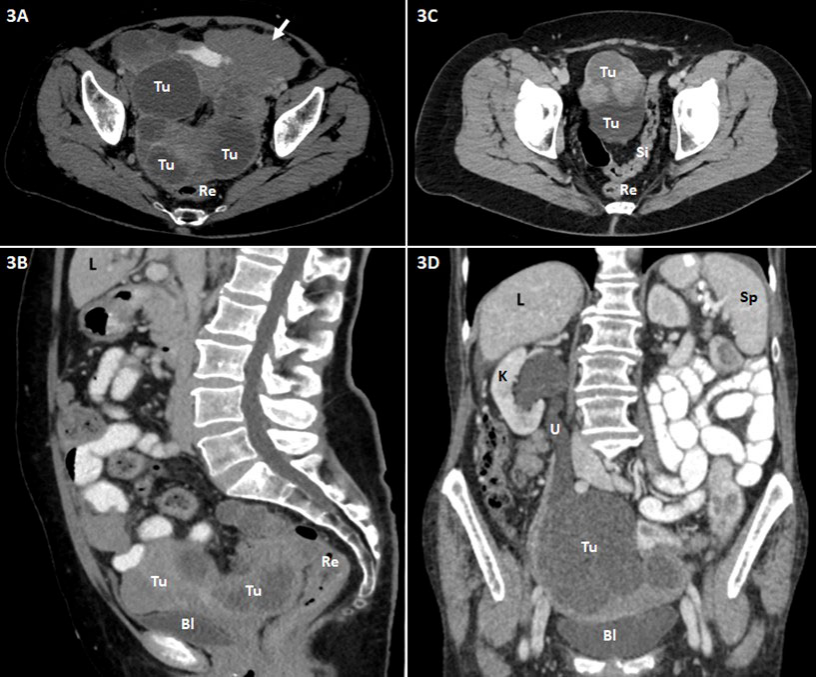
Abdominopelvic contrast-enhanced CT scans (A and C, axial images; B, sagittal reformatted image; D, coronal reformatted image) of patients with advanced ovarian carcinoma are shown. Bl, urinary bladder; L, liver; K, kidney; Re, rectum; Si, sigmoid colon; Sp, spleen; Tu, tumor (ovarian carcinoma); U, ureter. **(A, B)** Extensive tumor (Tu) in the pelvis consisting of large cystic and partly solid nodular parts. Close positional relationship and contact of the mass to the dorsally adjacent rectum (Re) and ventrally adjacent urinary bladder (Bl) with suspected rectal invasion and peritoneal involvement at the urinary bladder roof. Omental caking (short arrow) in the left side of the upper abdomen. **(C)** Massive tumor (Tu) in the pelvis including cystic and solid nodular parts. Close positional relationship and contact of the mass to the adjacent sigmoid colon (Si); tumor involvement of the latter is conceivable. **(D)** Large, predominantly cystic tumor mass (Tu) in the pelvis with involvement of the right ureter (U) and consecutive ipsilateral urinary retention and hydronephrosis of the right kidney (K).

**Figures 1**–**3** are representative examples of evaluated CT findings within our real-world cohort of patients.

### Small bowel carcinomatosis

As depicted in **Table 2**, there was a significant correlation between suspected small mesentery carcinomatosis in the preoperative CT scan and the intraoperative finding of a small bowel mesentery carcinomatosis (p = 0.001). However, 57 patients (54.3%) were deemed to be not evaluable in the preoperative CT scan regarding this issue. The negative predictive value (NPV) was 35%, and the positive predictive value (PPV) was only 7.14%. Surgery reports documented a small bowel mesentery carcinomatosis in 23 of 57 non-evaluable patients. The intraoperatively documented small bowel serosal carcinomatosis showed no significant correlation with the CT scan finding of a wall thickening of the small bowel (p = 0.08). The NPV was 87.67%, and the PPV was 25.59%. Small bowel carcinomatosis of the root was suspected in 48 cases, and in 55 cases, the root was deemed unsuspicious, and two cases were not evaluable. During surgery, 15 of 48 suspected patients showed carcinomatosis of the root of the small mesentery, and 5 of 56 unsuspected patients showed a mesentery root carcinomatosis reaching a significant correlation (p = 0.006). The NPV was 90.91%, but the PPV was only 31.35%.

Looking into the visibility of lymph nodes within the mesenteric root, we found 51 patients in total with numerous (>10) small mesenteric lymph nodes at the mesenteric root (number > 10, short axis diameter < 1 cm, oval configuration) and 53 patients without any visible mesenteric lymph node at the mesenteric root in the preoperative CT scan. One patient was deemed to be not evaluable using the preoperative CT scan.

**Figure 4:**
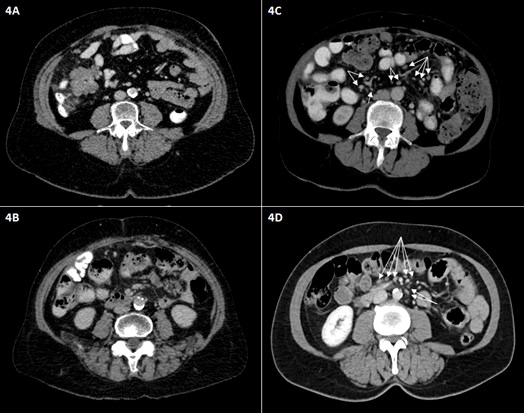
Representative axial intravenous contrast-enhanced CT scans of the middle abdomen. The absence of numerous small mesenteric lymph nodes is shown **(A, B)**; only small vessels in the mesenteric root are visible. **(C, D)** Other patients show multiple small mesenteric lymph nodes (<1 cm in short dimension) in the mesentery and mesenteric root (arrows).

The absence of multiple (>10) small mesenteric lymph nodes, defined as no visible mesenteric lymph nodes in the mesenteric root, as shown in **Figure 4**, was significantly more frequently observed in case of miliary carcinomatosis of the small bowel serosa, mesentery, or root, detected during surgery.

In detail, 40 of 67 patients without carcinomatosis of the mesentery showed numerous mesenteric root lymph nodes, while 27 of 38 patients with mesenteric carcinomatosis had no detectable mesenteric lymph node in the mesenteric root on the CT scan (p = 0.002), showing a PPV of 21.57% and an NPV of 50% for the detection of peritoneal carcinomatosis of the mesentery in the preoperative CT scan.

Of 21 patients with carcinomatosis of the mesenteric root, 18 showed no visible mesenteric lymph node, while 48 of 84 patients without carcinomatosis of the mesenteric root showed numerous mesenteric lymph nodes (p < 0.001), showing a PPV of 5.9% and an NPV of 66.7% for the detection of peritoneal carcinomatosis of the mesenteric root in the preoperative CT scan.

In 14 of 18 patients with small bowel mesenteric serosal carcinomatosis, detected during surgery, no mesenteric lymph nodes were detectable within the preoperative CT scan, while 47 of 87 patients without serosal carcinomatosis showed numerous mesenteric lymph nodes (p = 0.019), showing a PPV of 7.8% and an NPV of 74.1% for the detection of serosal peritoneal carcinomatosis in the preoperative CT scan.

**Figure 5:**
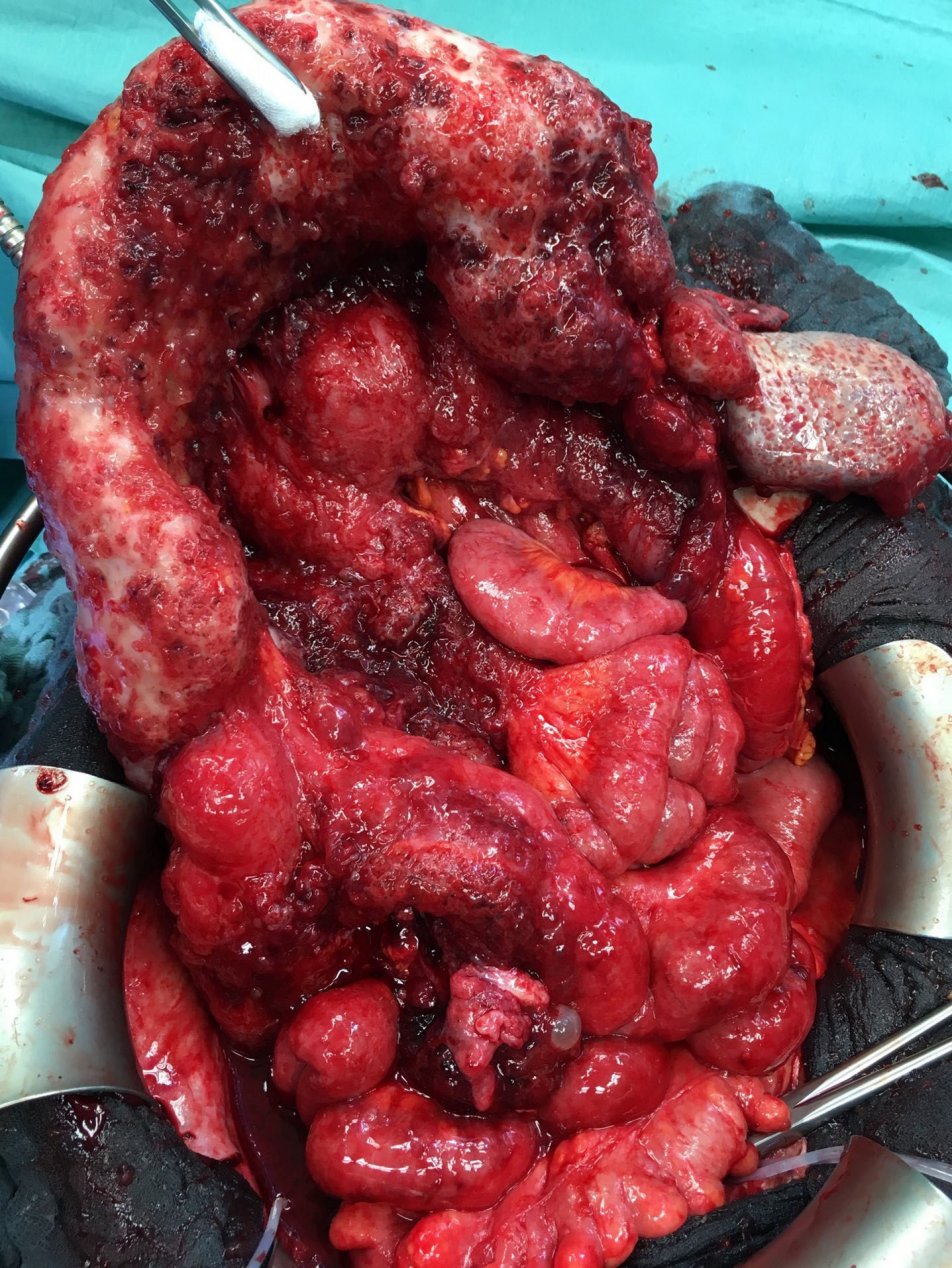
Intraoperative finding of peritoneal carcinomatosis of the mesenteric root.

In total, 40 patients had an intraoperatively documented carcinomatosis in at least one of the three above-mentioned locations (mesenteric root, small bowel mesentery, or small bowel serosa). In 28 of 40 patients, mesenteric lymph nodes were absent in the preoperative CT scan. In 12 patients, lymph nodes were present within the mesenteric root in the preoperative CT scan (p = 0.003), showing a PPV of 52.8% and an NPV of 76.5% for the detection of peritoneal carcinomatosis of the small bowel at the mesenteric root, the mesentery, or the serosa in the preoperative CT scan. **Figure 5** shows the intraoperative finding of carcinomatosis of the mesenteric root, the mesentery, and the serosa.

Large-volume ascites were found in 38 patients. Of these patients, 27 showed carcinomatosis of the small bowel in at least one of the above-mentioned locations (root, mesentery, or serosa). In 19 of 38 patients with large-volume ascites, complete cytoreduction was achieved.

Pathology reports in the case of large-volume ascites showed 34 high-grade serous, three high-grade endometrioid, and one clear cell histology.

An omental cake was seen in 33 patients. In 9 of 33 patients, the imaging showed no serosal or transmural transverse colon carcinomatosis, and in two cases, imaging was inconclusive regarding a serosal/transmural transverse colon infiltration. In further 10 patients, there was a transmural transverse colon infiltration by large tumor nodules originating from the colic mesentery in absence of an omental cake.

Cytoreduction was incomplete in 26 patients, and 21 of them showed miliary small bowel carcinomatosis (serosa, root, and/or mesentery), but in one patient, the irresectability was mainly due to involvement of the porta hepatis. In 17 of 21 patients, there were no small mesenteric lymph nodes visible. In total, small mesenteric lymph nodes were absent in 53 patients. In 28 of 53 patients, there was a small bowel carcinomatosis (serosa/root and/or mesentery) present.

### Regression analysis of all parameters with significant correlation between intraoperative findings and preoperative CT scan

In FIGO stage III and IV patients, the completeness of cytoreduction was compared to 13 variables that achieved significance with respect to imaging and intraoperative finding and the presence of no, little, or large-volume ascites. In the case of ascites in all four quadrants of the abdomen, considered large-volume ascites with the absence of numerous small mesenteric lymph nodes and peritoneal carcinomatosis of the transverse colon, the rate of complete cytoreduction was as low as 26.68%. In the case of a tumor-free transverse colon in the preoperative CT scan but large-volume ascites and absent mesenteric lymph nodes, the rate for complete cytoreduction was as low as 44.51% as seen in **Table 3**.

**Table 3:** Multivariate binary logistic regression analysis of 14 significant variables in FIGO IIIA–IVB patients.

Ascites 1. . .none 2. . .only pelvic ascites 3. . . .ascites in all 4 quadrants	Numerous small mesenteric lymph nodes 0. . .invisible 1. . .visible	Involvement of the transverse colon 1. . .yes 2. . .no	Rate of completely cytoreduced patients in total	Rate of completely cytoreduced patients in case of primary debulking surgery (PDS)	Rate of completely cytoreduced patients in case of interval debulking surgery (IDS)
1	0	1	68.82%	29.78%	68.23%
1	0	2	82.94%	64.95%	79.79%
1	1	1	89.5%	54.90%	88.37%
1	1	2	94.94%	84.14%	93.39%
2	0	1	56.15%	22.23%	55.79%
2	0	2	73.84%	55.53%	70.11%
2	1	1	83.19%	45.07%	81.70%
2	1	2	91.60%	78.19%	89.24%
3	0	1	26.68%	15.56%	24.23%
3	0	2	44.51%	44.79%	37.40%
3	1	1	58.44%	34.77%	53.11%
3	1	2	75.60%	69.96%	67.88%

The same group of patients was further distinguished into patients receiving neoadjuvant chemotherapy and interval debulking surgery (IDS) and patients receiving primary debulking surgery (PDS). In the case of IDS large-volume ascites, absent numerous mesenteric lymph nodes and peritoneal carcinomatosis of the transverse colon led to a complete cytoreduction rate of 24.23% and in the case of PDS to a complete cytoreduction rate of 15.66% as depicted in **Table 3**.

FIGO stage I and II patients were excluded from the multivariate analysis, as complete cytoreduction is always possible in this patient population.

## Discussion

In this study, the combination of large-volume ascites, peritoneal carcinomatosis of the transverse colon, and the absence of numerous small lymph nodes in the small bowel mesentery in the preoperative CT scans of the abdomen identified a group of patients where complete cytoreduction was achieved in less than 27% of the patients. Of course, optimal cytoreduction is a matter of tumor burden and surgical skill. When analyzing the optimal cytoreduction rate of ovarian cancer patients at our institution, we found that 75.2% of patients had no macroscopic visible residual tumor at the end of surgery. In the case of advanced disease (FIGO stage III and IV), the rate was 63.9%, consistent with recently published data (2, 10, 11, 15). The most frequent site of failure of optimal cytoreduction in our study was a carcinomatosis of the small bowel (mesentery, root, and serosa) accounting for almost 80% of all cases with residual tumors. Similar findings were previously described, concluding that the success of surgery regarding optimal cytoreduction in ovarian cancer patients depends on the presence or absence of PC in specific regions rather than only on the amount of PC in general (10, 11, 16).

While preoperative staging by computed tomography of the abdomen and thorax is by far the most common approach for presurgical evaluation due to its wide availability, the substantial underestimation of visceral small bowel peritoneal carcinomatosis is its major drawback (16, 17). The general pooled sensitivity and specificity for correct identification of region-based peritoneal carcinomatosis is 68% and 88% in ovarian and gastric cancers, respectively, depending on the size of the lesions and the presence of ascites (9).

As the intraoperatively generated PCI score shows low interobserver variability, several promising attempts were made to describe tumor load by a preoperative CT scan-based PCI score. It was limited by a general underestimation of the tumor burden in the small bowel and hepatoduodenal ligament (regions 2 and 9 to 12), which are the most likely locations for residual disease (10, 11, 14, 18–20).

The CT scan sensitivity decreases substantially in tumor sizes below 5 mm. In our experience, the size of 5 mm or less comprises the size of the single carcinomatosis nodule on the visceral peritoneum seen during surgery in most cases (16, 21). Therefore, the small bowel carcinomatosis itself, the most common location of residual disease, is usually not visible in the presurgical CT scan.

In this context, MRI (contrast-enhanced and diffusion-weighted imaging (DWI) images) is generally considered more accurate and sensitive, especially for the detection of liver metastases, perihepatic and serosal tumor nodules, and tumor implants on the hepatoduodenal and gastrohepatic ligament, diaphragm, and small intestinal wall. In contrast, results of recent studies showed that despite the highest sensitivity of MRI and the highest specificity of FDG-PET/CT, no significant differences were found between the three techniques (MRI, CT, and FDG-PET/CT) (22). Therefore, as the fastest, most economical, and widely available modality in daily practice and real life, CT is the examination of choice in particular when a stand-alone technique is needed. If inconclusive, PET/CT or MRI may offer additional insights. Whole-body FDG-PET/CT may be more accurate for a supradiaphragmatic metastatic extension. Despite advances in imaging techniques, neither DWI-MRI nor CT nor FDG-PET/CT seems to be superior in preoperative assessment of the surgical PCI in patients scheduled for upfront cytoreductive surgery for advanced-stage EOC patients (23).

As there are numerous patients with high tumor load but tumor-free small bowel and tumor-free hepatoduodenal ligaments, patients with the residual disease may represent a subgroup of epithelial ovarian cancer patients with increased tumorigenicity, which allows this unfavorable unresectable tumor spread pattern (24). In our experience, carcinomatosis of the small bowel was always accompanied by a high tumor load (PCI > 15). The absence of multiple mesenteric lymph nodes in the case of peritoneal carcinomatosis of the small bowel may be due to a decreased immune reaction. In this context, it is interesting to note that in triple-negative breast cancer patients, a missing germinal center formation in cancer-free lymph nodes is an indicator of a poorer prognosis. Therefore, this was considered a sign of a decreased systemic immune response (25). Furthermore, MRI in the case of Crohn’s disease remains unspecific regarding the proximal disease extension. However, the evaluation of the inflammation of small bowel mesentery lymph nodes shows the proximal disease extent despite unsuspicious bowel walls. Considering the visible inflammation in the case of an active Crohn’s disease, invisible mesenteric lymph nodes in the case of miliary small bowel carcinomatosis seem a noteworthy feature and a possible example of an immune escape of the tumor, leading to this unfavorable tumor spread (26). In addressing the problem of a diffuse tumor spread on the guts, three different clinical phenotypes of epithelial ovarian cancer patients were recently introduced, defining the diffuse tumor spread pattern within the rectosigmoid mesentery as the most lethal phenotype as compared to two other phenotypes with more localized disease and better survival outcomes (27).

Several studies reported diffuse peritoneal thickening, mesenteric disease, suprarenal lymph nodes, large-volume ascites, and carcinomatosis on the diaphragm or liver as significant markers in their final prediction model for complete cytoreduction (5, 21, 28, 29). The implementation of extensive upper abdominal surgery including diaphragm stripping, splenectomy, distal pancreatectomy, and resection of disease on the hepatoduodenal ligament made some of the above-mentioned markers less predictive for complete cytoreduction (10, 11, 15, 21, 28, 29).

Laparoscopy is an interesting evaluation tool in advanced ovarian cancer patients with positive prediction rates of 62% for cytoreduction to less than 1 cm of tumor rest (10, 30–33). To optimize that rate, different calculation models included the findings of CT scans, laparoscopy, and laparotomy, identifying a marker constellation of a PCI of 20 and more and bowel obstruction as significant for incomplete cytoreduction. As bowel obstruction is rather rare despite high tumor loads and reason for surgical intervention anyhow, this model may not be suitable for all patients in everyday business to prevent futile surgery (34).

Of course, there are limitations to our study. First of all, its retrospective nature always harbors the risk of bias. Second, there is a relatively high rate of patients treated with neoadjuvant chemotherapy due to the prior policy of the clinic to administer neoadjuvant chemotherapy in case of ascites of more than 500 cc.

The strength of our study compared to others is the definition of macroscopically no residual disease as an optimal cytoreductive outcome as residual disease regardless of whether size impacts prognosis more severely than any further available therapeutic tool (2, 12). Furthermore, we analyzed only radiological features, as we believe that incomplete cytoreduction due to a compromised performance status or ASA status as well as age comprises a different group of patients generally undergoing incomplete resection to avoid complications rather than because of an unfavorable tumor spread pattern (35). A third noteworthy feature is that we included only primary ovarian cancer patients, as prior surgery will affect the quality of the CT scan evaluation (16). A final strength of our study is that no further surgical intervention, anesthesia, or a new method of imaging was necessary to identify this unfavorable group of patients, which makes our finding applicable in the clinical day-to-day business (36).

To our knowledge, we are the first to describe the absence of multiple small bowel mesenteric lymph nodes as one of three markers predicting a very low chance for complete cytoreduction. As only one diagnostic tool was necessary for our finding, it is noteworthy for further prospective evaluation in larger cohorts and in combination with additional diagnostic techniques to encircle the group of patients without or with little benefit from surgery even better. Therefore, our finding may add valuable information for the decision between neoadjuvant chemotherapy or upfront debulking in ovarian cancer patients deemed fit enough for surgery. In our department, we implemented laparoscopy as an additional diagnostic tool in case of large-volume ascites, carcinomatosis of the transverse colon, and absent small bowel mesenteric lymph nodes in the preoperative CT scan. Different algorithms as additional preoperative abdominal MRI seem worth studying as well, in order to minimize any surgical intervention.

The key finding of our analysis is that the absence of numerous small mesenteric lymph nodes in the context of large-volume ascites and peritoneal carcinomatosis of the transverse colon in the presurgical CT scan of ovarian cancer patients are highly suspicious for miliary carcinomatosis of the small bowel and an unresectable tumor spread pattern.

## Data availability statement

The raw data supporting the conclusions of this article will be made available by the authors, without undue reservation.

## Ethics statement

The studies involving human participants were reviewed and approved by Ethics committee of the Faculty of Medicine at the University of Bonn, Germany. The patients/participants provided their written informed consent to participate in this study.

## Author contributions

EE and MM conceived and designed the analysis. MB, MM, and HS collected the data. FR and MS contributed data or analysis tools. MB and EE performed the analysis. EE wrote the paper. AM and MM edited the paper. All authors contributed to the article and approved the submitted version
